# Effect of antimicrobial de-escalation strategy on 14-day mortality among intensive care unit patients: a retrospective propensity score-matched cohort study with inverse probability-of-treatment weighting

**DOI:** 10.1186/s12879-023-08491-7

**Published:** 2023-08-04

**Authors:** Kai zhao, Zhengliang zhang, Ying liang, Yan wang, Yan cai

**Affiliations:** 1 Department of Pharmacy, the Second Affiliated Hospital of Xi’an Jiaotong University, Shaanxi 710004 Xi’an, China; 2 Department of Pharmacy, Northwest Women’s and Children’s Hospital, Shaanxi 710061 Xi’an, China; 3 Emergency Department, the Second Affiliated Hospital of Xi’an Jiaotong University, Shaanxi 710004 Xi’an, China; 4 Department of Medical Statistics, Air Force Medical University, Shaanxi 710032 Xi’an, China

**Keywords:** Antimicrobial de-escalation, Intensive care unit, Propensity score inverse probability of treatment weighting, Mortality

## Abstract

**Purpose:**

This study aimed to investigate the prevalence of antimicrobial de-escalation (ADE) strategy and assess its effect on 14-day mortality among intensive care unit patients.

**Methods:**

A single-center retrospective cohort study was conducted on patients admitted to the intensive care unit (ICU) with infectious diseases between January 2018 and December 2020. Patients were stratified into three groups based on the initial treatment regimen within 5 days of antimicrobial administration: ADE, No Change, and Other Change. Confounders between groups were screened using one-way ANOVA and Chi-square analysis. Univariate and multivariate analyses were performed to identify risk factors for 14-day mortality. Potential confounders were balanced using propensity score inverse probability of treatment weighting (IPTW), followed by multivariate logistic regression analysis to evaluate the effect of ADE strategy on 14-day mortality.

**Results:**

A total of 473 patients met the inclusion criteria, with 53 (11.2%) in the ADE group, 173 (36.6%) in the No Change group, and 247 (52.2%) in the Other Change group. The 14-day mortality rates in the three groups were 9.4%, 11.6%, and 21.9%, respectively. After IPTW, the adjusted odds ratio for 14-day mortality comparing No Change with ADE was 1.557 (95% CI 1.078–2.247, *P* = 0.0181) while comparing Other Change with ADE was 1.282(95% CI 0.884–1.873, *P* = 0.1874).

**Conclusion:**

The prevalence of ADE strategy was low among intensive care unit patients. The ADE strategy demonstrated a protective effect or no adverse effect on 14-day mortality compared to the No Change or Other Change strategies, respectively. These findings provide evidence supporting the implementation of the ADE strategy in ICU patients.

**Supplementary Information:**

The online version contains supplementary material available at 10.1186/s12879-023-08491-7.

## Introduction

Antimicrobial de-escalation (ADE) is an important strategy in antimicrobial stewardship programs (ASPs) aimed at reducing the use of high-class antibiotics, which are often prescribed initially to effectively treat infectious diseases [1]. The primary goals of ADE include curbing the emergence of antibiotic-resistant bacteria and striking a balance between providing adequate treatment and avoiding unnecessary antibiotic use [2, 3]. S Several clinical practice guidelines and expert consensus support the implementation of ADE in the intensive care unit (ICU) for patients with infectious diseases [4, 5]. For instance, the Surviving Sepsis Campaign guidelines [4] recommend daily assessment for the possibility of ADE, while a panel of experts strongly advocates for timely implementation of ADE in intensive care unit patients receiving antimicrobials [5]. Specifically, ADE is encouraged once the pathogens or susceptibilities are identified.

Currently, there is ongoing debate regarding the efficacy and safety of ADE in ICU patients. Some evidence suggests that ADE is associated with a reduction in mortality compared to the continuation of empirical treatment, as revealed in a previous systematic review [6]. Another expert review considered ADE to be a likely safe intervention in intensive care unit patients [7]. Several studies have also shown that ADE can lead to favorable treatment outcomes, such as shorter hospital stays, no adverse effects on mortality, and a low incidence of side effects [8–13]. However, there are conflicting findings as well. Leone et al. [14] conducted a randomized controlled trial and found that de-escalation of antibiotics might prolong ICU hospitalization, although it did not pose any risks to mortality compared to the continuation-treatment group. Furthermore, a Cochrane review based on 493 studies found insufficient evidence on the efficacy and safety of de-escalation of antimicrobial agents in adults with sepsis, severe sepsis, or septic shock [15]. Additionally, De Bus et al. [16] conducted a retrospective observational study and concluded that ADE should not be considered a safe strategy when broad-spectrum therapy is used empirically. However, Kitsios et al. [17] argued that De Bus’s results might have been influenced by uncontrolled confounding bias and urged caution in interpreting observational evidence. A methodological study examining the impact of ADE on mortality identified limitations in recent observational studies [18]. These studies adjusted for baseline admission characteristics as potential confounders, but this might be insufficient when accounting for confounding by indication. Another limitation was the failure to adjust for time-varying variables, which could introduce immortal time bias [19].

In this study, we proposed the use of inverse probability of treatment weighting (IPTW) as a method to address potential confounder bias related to time-varying variables. We conducted a single-center retrospective cohort study at a tertiary hospital in China to assess the impact of the ADE strategy on 14-day mortality in ICU patients. The primary objective was to investigate whether the ADE strategy influenced patient outcomes while accounting for potential confounding factors.

## Methods

### Study design

We conducted a single-center retrospective cohort study at the Second Affiliated Hospital of Xi’an Jiaotong University. The study included ICU patients who were screened for infectious diseases between January 2018 and December 2020. Detailed data were obtained by reviewing each patient’s medical records. Ethical considerations were a priority, and the study protocol received approval from the Biomedical Ethics Committee of Xi’an Jiaotong University (Approval No. 2021 − 971).

### Study population and selection criteria

Patients were included in the study based on specific inclusion criteria. To be eligible for enrollment, patients had to meet the following criteria: (i) diagnosed with infectious diseases, (ii) aged 18 years or older, (iii) admitted to the ICU for a duration of at least 48 h, (iv) received antibacterial treatment for a minimum of 24 h, and (v) had a survival time of at least 5 days. All patients received regular medical treatment as recommended in current guidelines.

### Data collection

During the study period, we designated the first day of antimicrobial administration as day 0 and followed patients until day 14. We recorded patient mortalities during their ICU stay and on day 14 after the initiation of antimicrobial treatment. In cases where patients were discharged from the ICU before day 14, we continued to follow them up until the 14th day.

Various data were collected for each patient, including: (i) Demographic information: sex, age, dates of ICU admission and discharge, admission and discharge diagnoses, underlying diseases, immunosuppression status, and histories of antibiotic exposure; (ii) Microbial data : We collected information on pathogen detection, including any identified pathogens during the course of the infectious disease; (iii) Treatment information: This encompassed data on Sequential Organ Failure Assessment (SOFA) scores, collected on day 0 and day 5, relapse of infection, duration of antimicrobial therapy, surgical status, mechanical ventilation, central venous catheterization, indwelling drainage tube, and hemodialysis; (iv) Medication regimen : We recorded details of empirical antimicrobial prescriptions. Admission diagnoses were classified according to the International Statistical Classification of Diseases (ICD-11) [20].

In our study, we classified patients into three groups based on the initial treatment regimen observed within 5 days following the first day of antimicrobial administration. The classification was as follows: ADE group, No Change group, and Other Change group. The ADE group was defined according to the statement provided by expert panels [5]. It included patients whose treatment regimen met the following criteria: (i) Replacing broad-spectrum antimicrobials with agents of a narrower spectrum or lower ecological impact, and (ii) Stopping components of an antimicrobial combination. The No Change group consisted of patients whose initial treatment regimen remained unchanged between day 0 and day 5, whereas the Other Change group was defined as patients whose treatment regimen met the following criteria: (i) Replacing narrow-spectrum antimicrobials with agents of a broader spectrum, and (ii) Replacing monotherapy with combination therapy or an increase in the number of antimicrobial agents.

### Statistical analysis

Continuous variables such as SOFA day0, SOFA day5, and delta SOFA were transformed into categorical variables using the interquartile range and median. Variables were presented as frequencies (percentages) for categorical variables. One-way ANOVA, Kruskal-Wallis rank sum and chi-square tests were conducted to identify potential confounders between the ADE, No Change, and Other Change groups. Univariate logistic regression analysis was performed to screen for factors that potentially influenced 14-day mortality. Significant variables from the univariate analysis were then included in a multivariate logistic regression model to further explore their independent effects on mortality. To balance the covariates between groups, propensity scores and associated weights were computed using the twang package in R. A generalized boosted model with 3000 regression trees was used to obtain optimal balance among the groups by calculating inverse probability weights based on the propensity scores. Hypothesis tests (t-test or chi-square test) and standardized mean differences were then performed to evaluate the effect of weights on each confounding factors [21]. An absolute standardized mean difference (ASMD) of less than 0.1 indicated good balance between the groups. Finally, the inverse probability of treatment weighted odds ratio (IPTW-OR) was calculated for each group using IPTW-adjusted logistic regression analysis. This provided the final effect estimates, taking into account the weights derived from the propensity scores. Odds ratios (ORs) greater than 1 indicated a risk factor for 14-day mortality compared to the ADE strategy, while ORs less than 1 indicated a protective effect. Statistical analyses were performed using SAS software (version 9.4) and mnps (multinomial propensity score) function in the twang package in R Statistical Software (version 3.4.2).

## Results

### Baseline characteristic

**Fig. 1:**
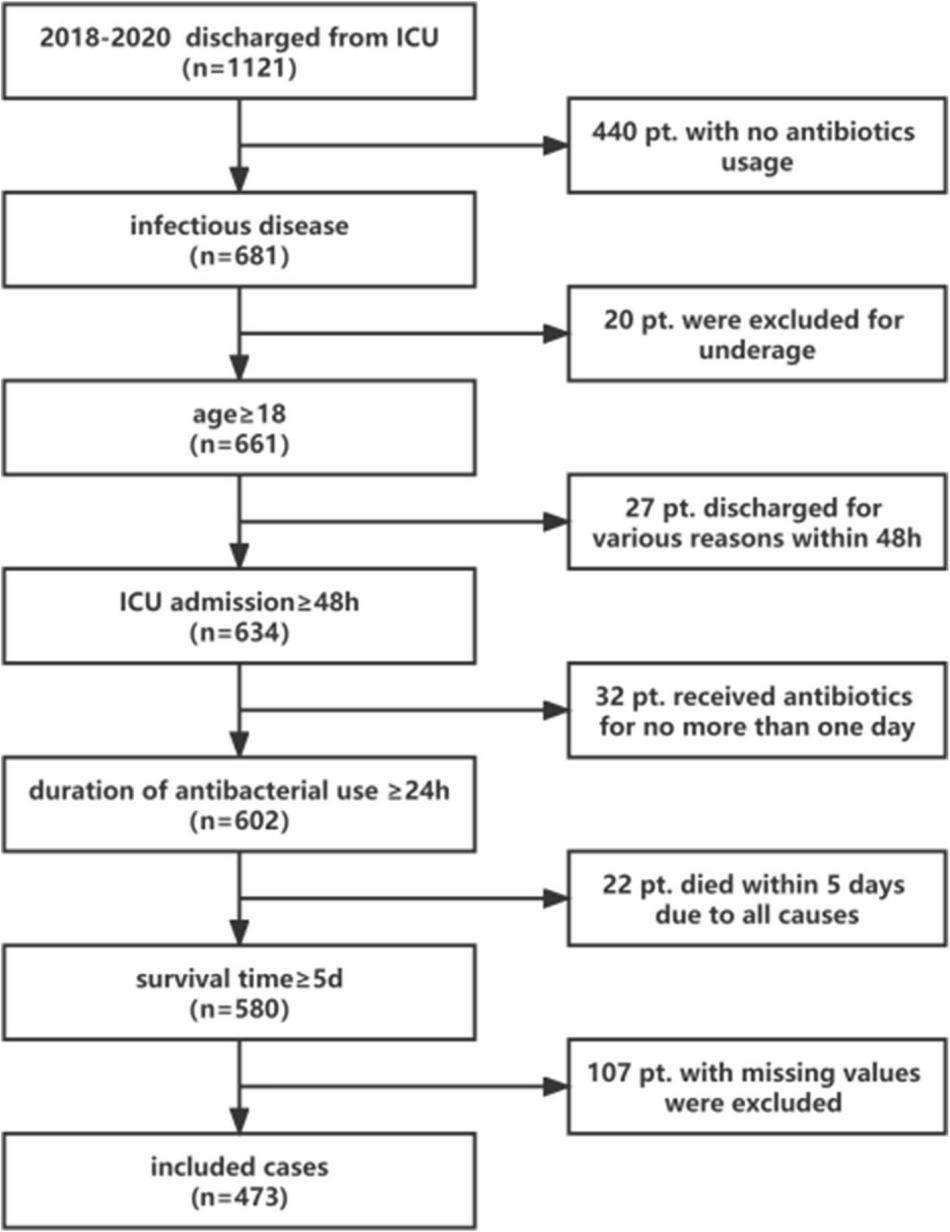
Flowchart of the inclusion/exclusion process. pt.: patients

Patients who met these criteria were enrolled in the study, resulting in a total of 473 patients included in the analysis (refer to Fig. 1). Table 1 displays the baseline characteristics of the 473 patients included in this study. Over half of the patients were elderly, with 53.1% being above 65 years of age. Among the enrolled patients, 58.8% (278/473) were male. The main admission diagnoses consisted of respiratory diseases (32.3%), circulatory diseases (17.3%), and neurological diseases (16.1%). The majority of patients (74.6%, *n* = 353) had underlying diseases, with an average of 2.13 diseases per patient. The most prevalent underlying conditions were cardiovascular diseases (65.7%), diabetes (31.4%), and cerebrovascular disease (30.0%). Approximately one-eighth of the patients (*n* = 60) had immunosuppression, with 64.3% of them having a history of antibiotic exposures in the three months prior to ICU admission. Among the patients, 11.2% (53/473) underwent de-escalation of therapy five days after initiating empirical antimicrobial treatment, while 36.6% (173/473) experienced no change and 52.2% (247/473) had other changes in their treatment regimens. There were no statistically significant differences observed between the groups regarding baseline factors.

**Table 1:** Baseline characteristics

	Total *n* = 473	ADE (*n* = 53)	No Change (*n* = 173)	Other Change (*n* = 247)	*P* value
Age ≥ 65	251(53.1%)	29(54.7%)	92(53.2%)	130(52.6%)	0.9619
Gender Male	278(58.8%)	35(66.0%)	99(57.2%)	144(58.3%)	0.5101
Admission Diagnosis^a^					
Septic shock or parasitic diseases	24(5.1%)	4(7.6%)	7(4.1%)	13(5.3%)	0.5854
Neoplasms	40(8.5%)	2(3.8%)	10(5.8%)	28(11.3%)	0.0565
Diseases of the blood or blood-forming organs	10(2.1%)	1(1.9%)	2(1.2%)	7(2.8%)	0.4968
Diseases of the immune system	10(2.1%)	2(3.8%)	2(1.2%)	6(2.4%)	0.4514
Endocrine, nutritional or metabolic diseases	39(8.3%)	4(7.6%)	19(11.0%)	16(6.5%)	0.2506
Mental, behavioral or neuro-developmental disorders	10(2.1%)	0	4(2.3%)	6(2.4%)	0.5231
Diseases of the nervous system	76(16.1%)	11(20.8%)	29(16.8%)	36(14.6%)	0.5134
Diseases of the circulatory system	82(17.3%)	12(22.6%)	32(18.5%)	38(15.4%)	0.3945
Diseases of the respiratory system	153(32.3%)	12(22.6%)	54(31.2%)	87(35.2%)	0.1905
Diseases of the digestive system	69(14.6%)	4(7.6%)	25(14.5%)	40(16.2%)	0.2694
Diseases of the skin	15(3.2%)	1(1.9%)	6(3.5%)	8(3.2%)	0.8445
Diseases of the musculoskeletal system or connective tissue	34(7.2%)	3(5.7%)	14(8.1%)	17(6.9%)	0.8057
Diseases of the genitourinary system	49(10.4%)	4(7.6%)	25(14.5%)	20(8.1%)	0.0850
Pregnancy, childbirth or the puerperium	4(0.9%)	1(1.9%)	0	3(1.2%)	0.2778
Other	48(10.2%)	6(11.3%)	15(8.7%)	27(10.9%)	0.7188
Underlying diseases	353(74.6%)	37(69.8%)	135(78.0%)	181(73.3%)	0.3777
Chronic pulmonary disease	67(19.0%)	6(16.2%)	29(21.5%)	32(17.7%)	0.4470
Chronic hepatic disease	48(13.6%)	3(8.1%)	24(17.8%)	21(11.6%)	0.1035
Chronic renal failure	69(19.5%)	7(18.9%)	28(20.7%)	34(18.8%)	0.7522
Diabetes mellitus	111(31.4%)	12(32.4%)	51(37.8%)	48(26.5%)	0.0567
Cardiovascular disease	232(65.7%)	27(73.0%)	87(64.4%)	118(65.2%)	0.8422
Solid tumor	68(19.3%)	4(10.8%)	21(15.6%)	43(23.8%)	0.1024
Hematologic malignancy	10(2.8%)	2(5.4%)	2(1.5%)	6(3.3%)	0.4514
Cerebrovascular disease	106(30.0%)	14(37.8%)	40(29.6%)	52(28.7%)	0.6700
Chronic gastrointestinal disease	5(1.4%)	0	2(1.5%)	3(1.7%)	0.7258
Other	36(10.2%)	4(10.8%)	18(13.3%)	14(7.7%)	0.1973
Immunosuppression status^b^	60(17.0%)	5(9.4%)	16(9.3%)	39(15.8%)	0.1054
Histories of antibiotic exposure^c^	304(64.3%)	40(75.5%)	108(62.4%)	156(63.2%)	0.1935

^*^P < 0.05, **P < 0.01, ***P < 0.001

^a^ according to the diagnostic criteria of ICD-11 and one patient may have one or more admission diagnoses

^b^ including acquired immune deficiency syndrome (AIDS) patients, solid organ transplantation, use of corticosteroids or immunosuppressants in the last 3 months before ICU admission

^c^ history of antibiotic use in the last 3 months before ICU admission

### Clinical characteristics and outcome

Table 2 presents the profiles of patient clinical characteristics and outcomes. Notably, 276 (58.4%) patients had positive culture results with multidrug-resistant (MDR) (135), non-MDR (96), and colonization or contamination (45), which differed significantly between the groups (*P* < 0.0001). Empirical antimicrobial regimens at admission also exhibited significant differences between groups (*P* < 0.0001), with most patients in the No Change and Other Change groups receiving monotherapy (78.0% and 71.3%, respectively), while nearly half of the patients in the ADE group were treated with two agents (*n* = 26, 49.1%). There were no observed cases of infection relapse during the 14-day period in any of the three groups. The duration of antimicrobial therapy and length of ICU stay differed significantly among the groups according to the Kruskal-Wallis test (*P* = 0.0054 and *P* = 0.0041, respectively). Results from the chi-square test indicated significant differences among the groups regarding 14-day mortality (*P* = 0.0066) and the cure rate before discharge (*P* < 0.0001).

**Table 2:** Clinical characteristics and outcome

	Total *n* = 473	ADE (*n* = 53)	No Change (*n* = 173)	Other Change (*n* = 247)	*P* value
SOFA day0^a^^, f^	5(3–7)	6(4–7)	5(3–8)	5(4–7)	0.8020
< = 5	248(52.4%)	26(49.1%)	94(54.3%)	128(51.8%)	0.7834
5 ~ 7	111(23.5%)	14(26.4%)	35(20.2%)	62(25.1%)	
> 7	114(24.1%)	13(24.5%)	44(25.4%)	57(23.1%)	
SOFA day5^a^^, f^	4(3–8)	4(3–7)	4(2–7)	4(4–9)	< .0001^***^
< = 4	186(39.3%)	26(49.1%)	80(46.2%)	80(32.4%)	0.0201^*^
4 ~ 8	184(38.9%)	19(35.9%)	62(35.8%)	103(41.7%)	
> 8	103(21.8%)	8(15.1%)	31(17.9%)	64(25.9%)	
Delta SOFA^b^^, f^					< .0001^***^
< 0	185(39.1%)	12(22.6%)	47(27.2%)	126(51.0%)	
> = 0	288(60.9%)	41(77.4%)	126(72.8%)	121(49.0%)	
Surgical status	88(18.6%)	9(17.0%)	26(15.0%)	53(21.5%)	0.2369
Pathogen detection^c^					< .0001^***^
Culture negative	148(31.3%)	28(52.8%)	58(33.5%)	62(25.1%)	
MDR^d^	135(28.5%)	12(22.6%)	28(16.2%)	95(38.5%)	
Non-MDR^d^	96(20.3%)	6(11.3%)	34(19.7%)	56(22.7%)	
Colonization or contamination	45(9.5%)	5(9.4%)	22(12.7%)	18(7.3%)	
No samples submitted or discharged	49(10.4%)	2(3.8%)	31(17.9%)	16(6.5%)	
Mechanical ventilation	355(75.1%)	33(62.3%)	121(69.9%)	201(81.4%)	0.0021^**^
Central venous catheterization	227(48.0%)	20(37.7%)	72(41.6%)	135(54.7%)	0.0089^**^
Indwelling drainage tube	173(36.6%)	13(24.5%)	59(34.1%)	101(40.9%)	0.0563
Hemodialysis	118(24.9%)	7(13.2%)	46(26.6%)	65(26.3%)	0.1109
Empirical antimicrobial prescription					< .0001^***^
Monotherapy	330(69.8%)	19(35.9%)	135(78.0%)	176(71.3%)	
2 agents	111(23.5%)	26(49.1%)	28(16.2%)	57(23.1%)	
> 2 agents	32(6.8%)	8(15.1%)	10(5.8%)	14(5.7%)	
Relapse of infection^e^	0(0%)	0(0%)	0(0%)	0(0%)	-
Duration of antimicrobial therapy (days)^f^	8(5–15)	9(5–14)	8(4–13)	9(6–16)	0.0054^**^
Length of ICU stay (days)^f^	8 (5–15)	9(5–14)	8(4–14)	9(7–16.5)	0.0041^**^
Cure rate	250(52.9%)	37(69.8%)	106(61.2%)	107(43.4%)	< .0001^***^
14-day mortality	79(16.7%)	5(9.4%)	20(11.6%)	54(21.9%)	0.0066^**^

^*^P < 0.05, ^**^P < 0.01, ^***^P < 0.001

^a^ Median SOFA day0 score was 5 (interquartile range: 3–7). Median SOFA day5 score was 4 (interquartile range: 3–8)

^b^ Delta SOFA is SOFA score on day 0 minus SOFA score on day 5 of infection

^c^ Between 3 days before day0 and day5. Detection results include metagenomics next generation sequencing (mNGS)

^d^ Emergence of pathogen including non-multidrug-resistant (MDR), multidrug-resistant (MDR), extensively drug-resistant (XDR) and pandrug-resistant (PDR). Criteria for defining MDR, XDR and PDR is based on an international expert proposal by Magiorakos et al. [22]

^e^ We observed no relapse in the 14-day period

^f^ Data are given as median (25%-75% range)

### Antimicrobial usage and distribution of pathogens

Overall, β-lactam/β-lactamase inhibitor combinations and carbapenems accounted for more than half of the anti-infection regimens (Supplement Table S1). Carbapenem was the most frequently used antimicrobial in the ADE group, representing less than a third of all prescriptions. Detailed information on all culture-positive pathogens can be found in Supplement Table S2 with a total of 312 strains were detected. Notably, 164 MDR strains were identified, with *Enterobacteriaceae* (85), *Acinetobacter* (28), *Enterococcus* (22), and *Staphylococcus* (17), accounting for a total of 92.7% of all MDR strains. The majority of the samples were sputum/BALF (59.3%), followed by other normally sterile body fluids (cerebrospinal fluid, ascitic fluid, joint fluid, and pleural effusion) (20.8%), blood (11.9%), and urine (8.0%) (Supplement Table S3).

### Univariate and multivariate analysis of prognostic factors of 14-day mortality

The risk factors associated with 14-day mortality were assessed through univariate and multivariate logistic regression analysis (Table 3). Delta SOFA, calculated as the difference between SOFA scores on day 0 and day 5, was included in the analysis. Univariate logistic regression analysis revealed that solid tumor, hematologic malignancy, Other Change strategy, delta SOFA, MDR, mechanical ventilation and central venous catheterization were identified as risk factors for 14-day mortality. The multivariate analysis indicated that solid tumor, hematologic malignancy, delta SOFA < 0, MDR, mechanical ventilation, and central venous catheterization remained as relative risk factors for 14-day mortality. However, in contrast to the univariate analysis results, the empirical antimicrobial prescription did not show statistical significance in the multivariate regression analysis. Compared to the ADE group, there was no statistically significant effect of the No Change strategy (OR, 0.991; 95%CI, 0.277–3.543; *P* = 0.9097) and Other Change strategy (OR, 0.894; 95%CI, 0.265–3.019; *P* = 0.7774) on 14-day mortality.

**Table 3:** Univariate and multivariate logistics regression analysis of 14-day mortality

Variable	Univariate analysis			Multivariate analysis		
	OR	95% CI	*P*	OR	95% CI	*P*
Underlying diseases						
Solid tumor	2.03	(1.11 ~ 3.713)	0.0215^*^	2.547	(1.226 ~ 5.293)	0.0122^*^
Hematologic malignancy	8.014	(2.207 ~ 29.1)	0.0016^**^	13.079	(2.619 ~ 65.310)	0.0017^**^
Empirical antimicrobial prescription						
Monotherapy	Ref	-	-	Ref	-	-
2 agents	0.904	(0.5 ~ 1.635)	0.7391	0.700	(0.346 ~ 1.416)	0.5642
> 2 agents	1.401	(0.577 ~ 3.399)	0.4565	0.794	(0.252 ~ 2.503)	0.9274
Antimicrobial strategy						
ADE	Ref	-	-	Ref	-	-
No Change	1.255	(0.447 ~ 3.523)	0.6664	0.991	(0.277 ~ 3.543)	0.9097
Other Change	2.686	(1.019 ~ 7.08)	0.0457^*^	0.894	(0.265 ~ 3.019)	0.7774
Delta SOFA^e^						
< 0	6.796	(3.89 ~ 11.872)	< .0001^***^	5.357	(2.876 ~ 9.979)	< .0001^***^
Pathogen detection^f^						
Culture negative	Ref	-	-	Ref	-	-
MDR^g^	3.041	(1.642 ~ 5.63)	0.0004^**^	2.750	(1.327 ~ 5.698)	0.0005^***^
Non-MDR^g^	1.233	(0.582 ~ 2.613)	0.5846	1.001	(0.422 ~ 2.371)	0.8592
Colonization or contamination	0.516	(0.145 ~ 1.839)	0.3077	0.404	(0.094 ~ 1.735)	0.0913
No samples submitted or discharged	0.642	(0.206 ~ 1.998)	0.4442	1.207	(0.337 ~ 4.319)	0.7951
Mechanical ventilation	16.064	(3.881 ~ 66.485)	0.0001^***^	15.328	(3.111 ~ 75.531)	0.0008^***^
Central venous catheterization	2.759	(1.65 ~ 4.614)	0.0001^***^	1.895	(1.047 ~ 3.430)	0.0347^*^

^*^P < 0.05, ^**^P < 0.01, ***P < 0.001; Ref., reference

^a^ According to the diagnostic criteria of ICD-11 and one patient may have one or more admission diagnoses

^b^ Including acquired immune deficiency syndrome (AIDS) patients, solid organ transplantation, use of corticosteroids or immunosuppressants in the last 3 months before ICU admission

^c^ History of antibiotic use in the last 3 months before ICU admission

^d^ Median SOFA day0 score was 5 (interquartile range: 3–7). Median SOFA day5 score was 4 (interquartile range: 3–8)

^e^ Delta SOFA is SOFA score on day 0 minus SOFA score on day 5 of infection

^f^ Between 3 days before day0 and day5. Detection results include metagenomics next generation sequencing (mNGS)

^g^ Emergence of pathogen including non-multidrug-resistant (MDR), multidrug-resistant (MDR), extensively drug-resistant (XDR) and pandrug-resistant (PDR). Criteria for defining MDR, XDR and PDR is based on an international expert proposal by Magiorakos et al. [21]

### Analysis of ADE strategy on 14-day mortality using IPTW

**Fig. 2:**
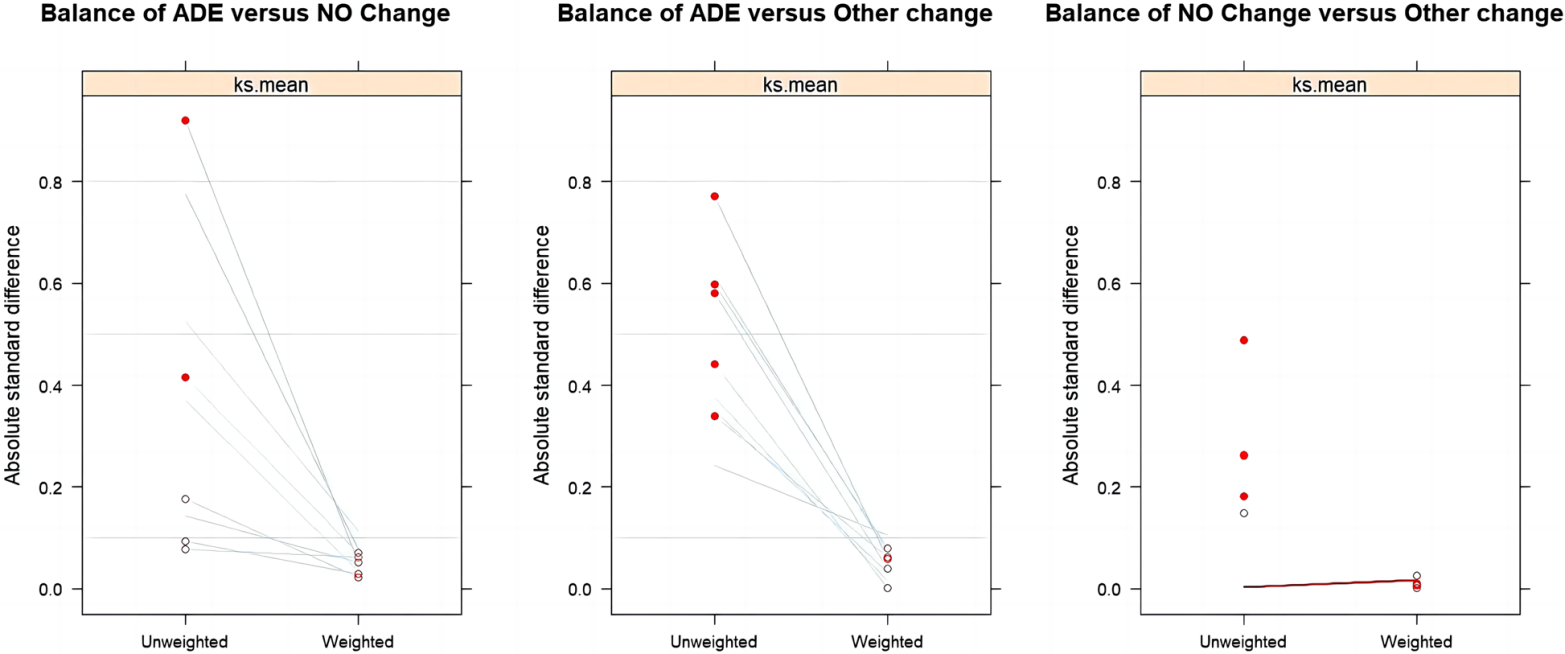
Absolute standardized mean differences among ADE, No Change and Other Change groups before and after IPTW weighting

To examine the impact of ADE on 14-day mortality, we utilized IPTW (Inverse Probability of Treatment Weighting) with a multigroup propensity score to account for potential confounding factors. As demonstrated in Table 2, significant differences were observed in empirical antimicrobial prescription, pathogen detection, mechanical ventilation, central venous catheterization, and delta SOFA among the groups. Therefore, these variables were included as confounding factors in the IPTW analysis. The balance in these covariates before and after weighting was assessed using ASMD. The results indicated that all covariates achieved sufficient balance across the different groups (ASMD < 0.10) (Fig. 2).

**Fig. 3:**
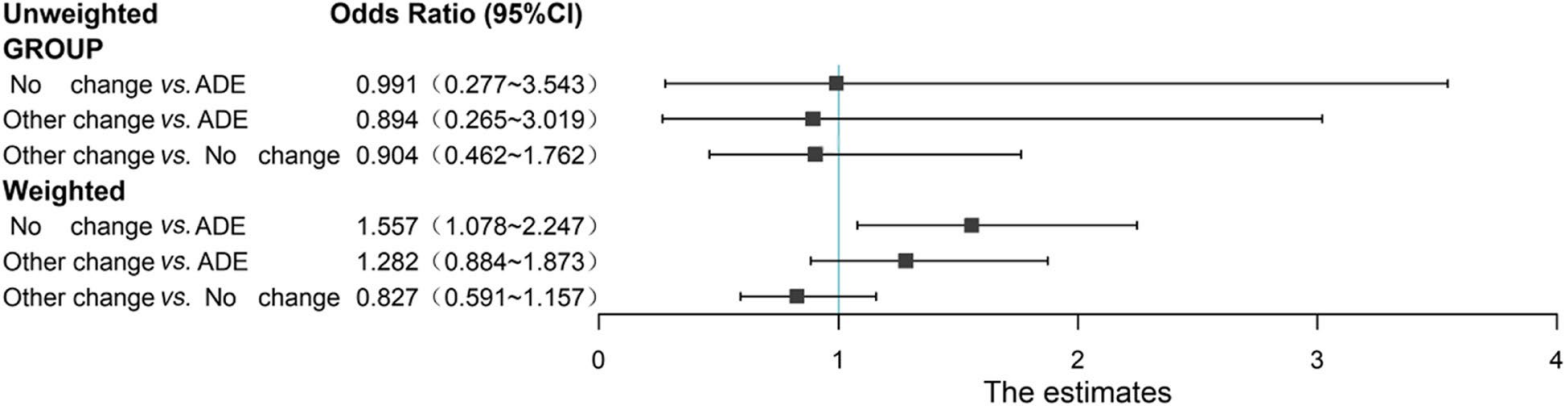
The unweighted and weighted odds ratio of 14-day mortality for three-group comparison

Following the weighting adjustment, the multivariate logistic regression analysis revealed a significant increase in the risk of 14-day mortality in the No Change group compared to the ADE group (OR, 1.557; 95%CI, 1.078–2.247; *P* = 0.0181). However, after weighting, there was no statistical difference between Other Change group and ADE group (OR, 1.282; 95%CI, 0.884–1.873; *P* = 0.1874), and the comparison of Other Change group and No Change group showed similar results (OR, 0.827; 95%CI, 0.591–1.157; *P* = 0.2677) (Table 4; Fig. 3).

**Table 4:** Multivariate and IPTW-adjusted logistic regression analysis of 14-day mortality between groups

Groups	Multivariate logistic regression			IPTW-adjusted analysis		
	OR	(95% CI)	*P*	OR	(95% CI)	*P*
No Change *vs.* ADE	0.991	(0.277 ~ 3.543)	0.9097	1.557	(1.078 ~ 2.247)	0.0181
Other Change *vs.* ADE	0.894	(0.265 ~ 3.019)	0.7774	1.282	(0.884 ~ 1.873)	0.1874
Other Change *vs.* No Change	0.904	(0.462 ~ 1.762)	0.7774	0.827	(0.591 ~ 1.157)	0.2677

## Discussion

Antimicrobial de-escalation therapy has been recommended by several guidelines as a treatment approach for ICU patients [23]. However, the impact of this strategy on patient outcomes remains controversial, despite the results of a multicenter non-blinded randomized noninferiority trial suggesting no effect on mortality [14, 16]. It is important to consider that there may be unmeasured confounding factors, including time-varying parameters that could influence the final conclusion. In our study, we utilized IPTW to identify and control for potential confounders that could affect both baseline characteristics and outcomes, thereby enabling a more precise evaluation of the effects of the ADE strategy on 14-day mortality in ICU patients.

To the best of our knowledge, only two previous studies have employed the IPTW method to control for potential confounders and assess the impact of the ADE strategy [24, 25]. However, these studies had their own limitations, such as the absence of measurable time-dependent confounders or residual confounding related to the short timing of ADE.

In our study, we conducted a single-center retrospective cohort study using propensity score inverse probability of treatment weighting. We made the assumption that there were no unmeasured confounders [26]. All potential confounding factors were initially recorded based on their clinical significance. Instead of using the Delphi Method described in De Bus’ study [25], we performed one-way ANOVA analysis to identify statistically significant variables, aiming to avoid potential limitations. Confounding factors were categorized into two groups: time-fixed factors, such as clinical treatment and interventions, and time-varying factors, including pathogen culture results and delta SOFA. These time-varying factors had an impact on both treatment outcomes and the decision to implement the ADE strategy.

To address the confounding factors between groups, we employed propensity score inverse probability of treatment weighting. Our analysis revealed that the ADE strategy appeared to have a protective effect on 14-day mortality compared to the No Change strategy. This suggests that implementing the ADE strategy may be a favorable option for patients in the No Change group. However, despite controlling for confounders, the ADE strategy did not show any apparent protective effects on mortality compared to the Other Change strategy. Therefore, in patients with uncontrolled infections or rapid disease progression, it may be more appropriate to consider an escalation strategy or other changes based on the patient’s condition rather than implementing the ADE strategy.

In our study, the ADE strategy was implemented in only 11.2% (*n* = 53) of all ICU patients, which is consistent with the findings of a recent study reporting a low ADE rate of 16.1% [25]. There are several possible reasons for the low prevalence of ADE in our study. Firstly, the definition of the timing of ADE could influence the ADE rates. If the ADE time-window is expanded to 7 days, the ADE rate could increase to 23% [25]. Secondly, the choice of specific spectrum of initial antimicrobials or combinations may impact the implementation of the ADE strategy. Broad-spectrum antibiotics and combination therapy are more likely to result in ADE. In our study, a higher proportion of patients in the No Change (135/173) and Other Change (176/247) groups received monotherapy and narrow-spectrum or weaker activity antibiotics such as third-generation cephalosporins and quinolones, making it difficult for further de-escalation. Another important factor affecting antibiotic regimens is the culture results. For intensive care unit patients, identification of non-MDR or negative culture results may lead to early initiation of target-directed therapy or discontinuation of antimicrobial treatment. In our study, we observed a higher proportion of negative culture results, with ADE representing a significant portion (52.8%). Conversely, MDR culture results often indicate disease progression and require an escalation strategy for antibiotics. Our results demonstrated that *Enterobacteriaceae* accounted for the highest proportion of MDR strains, with 38.5% of patients in the Other Change group exhibiting MDR. These findings align with similar results reported in the DIANA study, where the Other Change group had a higher proportion of MDR (19.6%, 63/321) [25].

The evaluation of clinical outcomes commonly includes the use of 28-day mortality [27, 28]. However, several factors beyond control, such as hospital transfers, accidental trauma, unsanitary environments, and non-reviewed cases, among others, can influence mortality rates during the 28-day follow-up period. In some studies focusing on septic shock patients, 14-day mortality has been used as an indicator for prognostic prediction [29]. In our study, both the length of ICU stay and the duration of antimicrobial therapy fell within the 5–15 day range, making 14-day mortality a suitable clinical outcome for analysis during IPTW.

This study is subject to several limitations. Firstly, the definition of ADE was based on statements from expert panels, which is classified as low-quality evidence [5]. A lack of consensus among studies included in a previous meta-analysis on the definition of ADE further complicates the issue [23]. Secondly, the majority of culture results in our study were obtained between the 3rd and 5th day. As a result, we defined the timing of the ADE strategy as a period from day 0 to day 5 after initiating the empirical antimicrobial regimen. However, other studies have defined the timing of ADE strategy from day 0 to day 3 or 4 [25, 28]. The variability in defining the timing of ADE strategy may affect the proportion of patients in the ADE group. Thirdly, this study focused solely on the effect of ADE strategy on 14-day mortality in ICU patients. The impact of ADE on other clinical outcomes, such as antimicrobial usage, length of hospital stay, and incidence of antimicrobial resistance, has not been assessed. Fourthly, we used only SOFA score, but not other severity scores like Acute Physiology and Chronic Health Evaluation (APACHE), Simplified Acute Physiology Score (SAPS), or Mortality Probability Model (MPM) to assess the severity of illness in ICU patients. Given their wide utilization, the assessment in ICU patients in this study seemed not very accurate due to the failure to use other scoring systems. Lastly, being a single-center retrospective cohort study, the generalizability of our findings may be limited. Therefore, conducting a large-scale multicenter study with comprehensive analysis of clinical outcomes would help further validate our conclusions.

## Conclusion

In conclusion, our study found that the ADE strategy had a low incidence of 11.2% among ICU infectious patients. It was associated with a lower 14-day mortality compared to the No Change strategy, and no significant difference compared to the Other Change strategy. However, to fully assess the effectiveness of the ADE strategy in ICU patients, additional research is required to evaluate its impact on a wider range of clinical outcomes.

### Supplementary Information

**Additional file 1**

## Data Availability

Underlying research data and materials can be accessed by contacting the corresponding author.
