# Supplementary material for: Effect of antimicrobial de-escalation strategy on 14-day mortality among intensive care unit patients: a retrospective propensity score-matched cohort study with inverse probability-of-treatment weighting
Source: BMC Infect Dis. 2023 Aug 4;23:508. doi: 10.1186/s12879-023-08491-7 (PMC10401733; doi:10.1186/s12879-023-08491-7)
Supplement: Supplementary file 1 — Additional file 1 [file 12879_2023_8491_MOESM1_ESM.docx]

Table S1 Initial antimicrobials

| Overall | Total  n=473 | ADE n=53  (11.2%) | No Change  n=173  (36.6%) | Other Change  n=247  (52.2%) |
| --- | --- | --- | --- | --- |
| β-lactam/β-lactamase inhibitor combinations | 197(30.1%) | 20(20.8%) | 78(35.3%) | 99(29.4%) |
| Carbapenems | 176(26.9%) | 28(29.2%) | 66(29.9%) | 82(24.3%) |
| Third-generation cephalosporins^a^ | 41(6.3%) | 4(4.2%) | 12(5.4%) | 25(7.4%) |
| Linezolid | 39(6.0%) | 9(9.4%) | 16(7.2%) | 14(4.2%) |
| Glycopeptides | 38(5.8%) | 9(9.4%) | 9(4.1%) | 20(5.9%) |
| Quinolones | 38(5.8%) | 6(6.3%) | 9(4.1%) | 23(6.8%) |
| Azoles | 35(5.4%) | 6(6.3%) | 14(6.3%) | 15(4.5%) |
| Tigecycline | 21(3.2%) | 5(5.2%) | 3(1.4%) | 13(3.9%) |
| Nitroimidazoles | 15(2.3%) | 2(2.1%) | 2(0.9%) | 11(3.3%) |
| Echinocandins | 14(2.1%) | 3(3.1%) | 5(2.3%) | 6(1.8%) |
| First-generation cephalosporins | 11(1.7%) | 1(1.0%) | 3(1.4%) | 7(2.1%) |
| Aminoglycosides | 8(1.2%) | 1(1.0%) | 1(0.5%) | 6(1.8%) |
| Second-generation cephalosporins^b^ | 7(1.1%) | 1(1.0%) | 2(0.9%) | 4(1.2%) |
| Sulfonamide | 5(0.8%) | 0 | 0 | 5(1.5%) |
| Penicillins | 4(0.6%) | 0 | 1(0.5%) | 3(0.9%) |
| Fosfomycin | 2(0.3%) | 0 | 0 | 2(0.6%) |
| Azithromycin | 1(0.2%) | 0 | 0 | 1(0.3%) |
| Doxycycline | 1(0.2%) | 1(1.0%) | 0 | 0 |
| Amphotericin B liposomal | 1(0.2%) | 0 | 0 | 1(0.3%) |
| Monotherapy—top 3 | n=330 | n=19 | n=135 | n=176 |
| β-lactam/β-lactamase inhibitor combinations | 158(47.9%) | 11(57.9%) | 73(54.1%) | 74(42.1%) |
| Carbapenems | 89(27.0%) | 4(21.1%) | 38(28.2%) | 47(26.7%) |
| Third-generation cephalosporins | 33(10.0%) | 2(10.5%) | 11(8.2%) | 20(11.4%) |
| Combination therapy—top 5 | n=143 | n=34 | n=38 | n=71 |
| Carbapenems | 87(60.8%) | 24(70.6%) | 28(73.7%) | 35(49.3%) |
| β-lactam/β-lactamase inhibitor combinations | 39(27.3%) | 9(26.5%) | 5(13.2%) | 25(35.2%) |
| Glycopeptides | 37(25.9%) | 8(23.5%) | 9(23.7%) | 20(28.2%) |
| Linezolid | 36(25.2%) | 9(26.5%) | 14(36.8%) | 13(18.3%) |
| Azoles | 34(23.8%) | 6(17.7%) | 14(36.8%) | 14(19.7%) |

^a^ including Latamoxef; ^b^ including Cefmetazole

| Table S2 Distribution and resistance of the pathogens | | | | | |
| --- | --- | --- | --- | --- | --- |
| Pathogen classification | pathogen detection | ADE | No Change | Other Change | total |
| *Staphylococcus spp.* | MDR | 1 | 4 | 12 | 17 |
|  | Non-MDR | 1 | 2 | 5 | 8 |
|  | COC | 0 | 5 | 2 | 7 |
| *Enterococcus spp.* | MDR | 1 | 4 | 17 | 22 |
|  | Non-MDR | 0 | 2 | 2 | 4 |
|  | COC | 1 | 2 | 2 | 5 |
| *Enterobacteriaceae* | MDR | 9 | 17 | 59 | 85 |
|  | Non-MDR | 1 | 8 | 6 | 15 |
|  | COC | 1 | 3 | 3 | 7 |
| *Pseudomonas aeruginosa* | MDR | 1 | 3 | 5 | 9 |
|  | Non-MDR | 1 | 2 | 5 | 8 |
|  | COC | 0 | 0 | 0 | 0 |
| *Acinetobacter spp.* | MDR | 5 | 3 | 20 | 28 |
|  | Non-MDR | 0 | 5 | 1 | 6 |
|  | COC | 1 | 2 | 2 | 5 |
| *Fungi* | MDR | 0 | 0 | 0 | 0 |
|  | Non-MDR | 1 | 12 | 28 | 41 |
|  | COC | 2 | 11 | 9 | 22 |
| Others^a^ | MDR | 0 | 1 | 2 | 3 |
|  | Non-MDR | 2 | 4 | 11 | 17 |
|  | COC | 1 | 0 | 2 | 3 |
| Total | MDR | 17 | 32 | 115 | 164 |
|  | Non-MDR | 6 | 35 | 58 | 99 |
|  | COC | 6 | 23 | 20 | 49 |
| COC: colonization or contamination; MDR: multidrug-resistant  ^a^ including *Stenotrophomonas maltophilia*, *Streptococcus pneumoniae*, *Haemophilus influenzae*, *Actinobacteria*, *Achromobacter xylosoxidans*, *Pseudomonas luteola*, *Shewanella alga*, [*mycobacterium tuberculosis*](http://dict.youdao.com/w/mycobacterium%20tuberculosis/#keyfrom=E2Ctranslation), *Kocuria rhizophila*. | | | | | |

Table S3 Sample source

| Sample source | pathogen detection | ADE | No Change | Other Change | Total |
| --- | --- | --- | --- | --- | --- |
| Sputum/BALF | MDR | 10 | 15 | 53 | 78 |
|  | Non-MDR | 4 | 26 | 42 | 72 |
|  | COC | 4 | 15 | 16 | 35 |
| Blood | MDR | 3 | 5 | 17 | 25 |
|  | Non-MDR | 1 | 1 | 6 | 8 |
|  | COC | 0 | 2 | 2 | 4 |
| Other normally  sterile body fluid^a^ | MDR | 3 | 8 | 33 | 44 |
|  | Non-MDR | 1 | 7 | 8 | 16 |
|  | COC | 2 | 3 | 0 | 5 |
| Urine | MDR | 1 | 4 | 12 | 17 |
|  | Non-MDR | 0 | 1 | 2 | 3 |
|  | COC | 0 | 3 | 2 | 5 |
| BALF: bronchoalveolar lavage fluid; MDR: multidrug-resistant; COC: colonization or contamination.  ^a^ cerebrospinal fluid, ascitic fluid, joint fluid and pleural effusion | | | | | |
